# Potential for Prebiotics as Feed Additives to Limit Foodborne *Campylobacter* Establishment in the Poultry Gastrointestinal Tract

**DOI:** 10.3389/fmicb.2019.00091

**Published:** 2019-01-31

**Authors:** Sun Ae Kim, Min Ji Jang, Seo Young Kim, Yichao Yang, Hilary O. Pavlidis, Steven C. Ricke

**Affiliations:** ^1^Department of Food Science and Engineering, Ewha Womans University, Seoul, South Korea; ^2^Department of Poultry Science, University of Arkansas, Fayetteville, AR, United States; ^3^Diamond V, Cedar Rapids, IA, United States; ^4^Center for Food Safety and Department of Food Science, University of Arkansas, Fayetteville, AR, United States

**Keywords:** *Campylobacter*, poultry, prebiotics, gastrointestinal tract, synbiotics

## Abstract

*Campylobacter* as an inhabitant of the poultry gastrointestinal tract has proven to be difficult to reduce with most feed additives. In-feed antibiotics have been taken out of poultry diets due to the negative reactions of consumers along with concerns regarding the generation of antibiotic resistant bacteria. Consequently, interest in alternative feed supplements to antibiotics has grown. One of these alternatives, prebiotics, has been examined as a potential animal and poultry feed additive. Prebiotics are non-digestible ingredients by host enzymes that enhance growth of indigenous gastrointestinal bacteria that elicit metabolic characteristics considered beneficial to the host and depending on the type of metabolite, antagonistic to establishment of pathogens. There are several carbohydrate polymers that qualify as prebiotics and have been fed to poultry. These include mannan-oligosaccharides and fructooligosaccharides as the most common ones marketed commercially that have been used as feed supplements in poultry. More recently, several other non-digestible oligosaccharides have also been identified as possessing prebiotic properties when implemented as feed supplements. While there is evidence that prebiotics may be effective in poultry and limit establishment of foodborne pathogens such as *Salmonella* in the gastrointestinal tract, less is known about their impact on *Campylobacter*. This review will focus on the potential of prebiotics to limit establishment of *Campylobacter* in the poultry gastrointestinal tract and future research directions.

## Introduction

*Campylobacter* is a prevalent foodborne pathogen in poultry such as chicken and turkey. It causes foodborne disease in humans (campylobacteriosis) due to consumption of contaminated poultry products, thus constituting a major public health issue (Sahin et al., 2002; Newell and Fearnley, 2003). Control of *Campylobacter* in poultry to improve microbiological safety is a primary concern for consumers and government food safety agencies (Lin, 2009). Traditionally, antibiotics have been widely utilized for improving growth performance in poultry; however, the word ‘antibiotic’ provokes a negative reaction from consumers and using antibiotics could also lead to the potential generation of antibiotic resistant pathogenic bacteria, thus the routine supplementation of antibiotics into poultry feeds has become less prevalent over time (Edens, 2003; Jones and Ricke, 2003; Ferket, 2004; Dibner and Richards, 2005; Griggs and Jacob, 2005).

As a result of the shift away from antibiotic supplementation there has been a tremendous growth in research and implementation of effective alternative control methods using a wide array of approaches including hygiene and biosecurity farming practices, drinking water treatments, chemical feed additives, bacteriophage application, vaccination, passive immunization, competitive exclusion cultures, host genetic selection strategies, bacteriophage therapy, and bacteriocin application (Tsubokura et al., 1997; Mead, 2000; Newell and Wagenaar, 2000; Boyd et al., 2005; Carrillo et al., 2005; Cole et al., 2006; Wagenaar et al., 2006; de Zoete et al., 2007; Li et al., 2008; Lin, 2009; Buckley et al., 2010; Scupham et al., 2010; Skånseng et al., 2010; Svetoch and Stern, 2010; Van Gerwe et al., 2010a; Hermans et al., 2011a,b; Layton et al., 2011; Sibanda et al., 2018). Given the ability of *Campylobacter* to readily establish in the poultry gastrointestinal tract (GIT) of poultry (Indikova et al., 2015), an obvious target for limiting its proliferation are feed additives that serve as mitigation agents when introduced to the GIT of poultry. These would include inhibitory agents such as botanicals, organic acids and bacteriophage and colonization preventative biologicals such as prebiotics and probiotics. In practice, prevention of *Campylobacter* colonization by probiotics and prebiotics may prove more difficult than initially perceived since *Campylobacter* appear to be extensively interconnected with the indigenous microbiota of the poultry GIT (Indikova et al., 2015). While both approaches have been considered as potential control measures, the focus in this review will be on prebiotics as a means to alter or shift the composition of the already established poultry GIT microbiota and the resulting impact on *Campylobacter* populations.

Prebiotics have also been considered as one of the effective methods to increase the beneficial bacteria within the microbiota in the GIT of various food animal species, including chickens, as well as limit foodborne pathogens in the GIT (Flickinger et al., 2003; Patterson and Burkholder, 2003; Callaway and Ricke, 2011; Hermans et al., 2011b; Hutkins et al., 2016; Gibson et al., 2017; Ricke, 2018; Santovito et al., 2018). Prebiotics also appear to be generally effective in poultry and reduce colonization of foodborne pathogens such as *Salmonella* in the GIT of poultry (Ricke, 2015; Roto et al., 2015; Micciche et al., 2018). However, much less is known on the effectiveness of prebiotics to limit *Campylobacter* establishment in poultry. The specific aim of the present review is to provide an overview of *Campylobacter* in the poultry GIT along with the definition of prebiotics and their subsequent effects on the GIT microbiota. This will be accompanied by a discussion on the recent findings on the impact of prebiotics as feed supplements on *Campylobacter* in the poultry GIT, and offer potential directions for future research.

## *Campylobacter* in the Poultry GIT

Much of the focus on controlling *Campylobacter* spp. is on decreasing contamination on meat and skin of poultry (Kudirkienë et al., 2013; Rosenquist et al., 2013) while relatively limited information is available detailing the *Campylobacter* prevalence or populations in preharvest poultry production and the mitigation efforts associated with management on the farm (Sibanda et al., 2018). For feed amendments such as prebiotics to be successful it is important to identify the sites in the poultry GIT where *Campylobacter* colonization is most likely to occur. Representative studies reporting *Campylobacter* prevalence and their population levels in the poultry GIT are listed in Table 1. Since *Campylobacter* colonization usually occurs in the lower GIT particularly in the ceca (Beery et al., 1988; Jeffrey et al., 2001; Fernandez et al., 2000), historically most of the research efforts have been focused on characterization and mitigation strategies associated with *Campylobacter* and the lower GIT of poultry. However, there is growing evidence that the upper compartments of the poultry GIT, particularly the crop, may also play an important role.

**Table 1:** *Campylobacter* in the poultry gastrointestinal tract.

Poultry	Sample	Sample number	Prevalence of *Campylobacter*	*Campylobacter* spp. counts (log CFU/g)	Reference
Market-age broiler chickens	Ceca	240	9 samples positive (3.8%)	–	Byrd et al., 1998
	Crop	359	224 samples positive (62.4%)	–	
New York-dressed broiler	Ceca	128 (32 birds ^∗^ 4 repetition)	128 samples positive (100%) with direct plating 81 sample positive (63%) after enrichment	6.8	Musgrove et al., 2001
	Crop	128 (32 birds ^∗^ 4 repetition)	122 samples positive (95.3%) with direct plating 127 samples positive (99.2%) after enrichment	3.7	
Market-weight turkey	Ceca	84	2 samples *C. jejuni* positive (2.1%)	–	Wesley et al., 2005
			96 samples *C. coli* positive (100%)	–	
	Crop	96	11 samples *C. jejuni* positive (13.1%)	–	
			61 samples *C. coli* positive (72.6%)	–	
Turkey during slaughtering	Ceca at evisceration step	30 (collected on July)	30 samples positive (100%)	6.0	Bily et al., 2010
		30 (collected on Sep)	22 samples positive (73.3%)	2.1	
		30 (collected on Oct)	30 samples positive (100%)	7.2	
		30 (collected on Nov)	30 samples positive (100%)	6.0	
Chicken	Ceca	24	–	8.5	Van Gerwe et al., 2010b
	Crop	23	–	4.8	
Chicken	Gizzard	4 male	–	2.5	Yusrizal and Chen, 2003
		4 female	–	2.5	
	Small Intestine	4 male	–	3.6	
		4 female	–	3.8	
	Large intestine	4 male	–	5.3	
		4 female	–	5.4	
	Cecal intestine	4 male	–	5.3	
		4 female	–	4.4	


Byrd et al. (1998) investigated the prevalence of *Campylobacter* in crops (*n* = 359) and ceca (*n* = 240) of market-age broiler chickens using Campy-Ceflex plates after Bolton broth enrichment and reported that the percentages of *Campylobacter*-positive in crops (224/359: 62.4%) were higher than that of the ceca (9/240: 3.8%). They concluded that *Campylobacter* in the crop may be a critical control point to minimize entry of *Campylobacter* into the poultry processing plant and to reduce their contamination in broilers. The higher incidence in the crop is somewhat surprising compared to the results from other studies. The authors suggested that the higher incidence in the crop may be related to the prolonged feed withdrawal required for broiler production in the United States prior to being transported to the processing plant. In addition, it must be noted that these results represent the percentage of birds positive for *Campylobacter* and not necessarily the total numbers of *Campylobacter* in the crop and ceca.

Musgrove et al. (2001) studied *Campylobacter* in the ceca and crops of 32 New York-dressed broilers (*n* = 128, 32 birds with four repetitions) collected from commercial processing plants with and without enrichment. The 128 ceca (100%) and 122 (95.3%) crops were contaminated with *Campylobacter* when they were identified by direct plating on Campy-Cefex agar plates. After enrichment, detection rate of *Campylobacter* in crops was increased (122/128: 99.2%) while enrichment resulted in significantly fewer positive samples from the ceca (81/128: 63%). The authors suggested that the reason for the decrease of 36.7% in the detection rate of *Campylobacter* in ceca was due to being subjected to enrichment compared to direct plating. This may be attributed to the fact that there are more bacterial species in the ceca than in the crop with higher numbers of total bacteria and thus *Campylobacter* species grow more slowly than other bacterial species along with poor competition ability in its intestinal niche (Musgrove et al., 2001). The contamination level in the ceca (6.8 log CFU/g) was twice as much as crops (3.6 log CFU/g) (Musgrove et al., 2001).

Prevalence of *C. jejuni* and *C. coli* on market-weight turkeys was studied by Wesley et al. (2005). A total of 84 crops and 96 ceca were tested (enrichment in blood-free enrichment broth, plating on Campy-Cefex agar, and confirmation of presumptive colonies with a multiplex PCR) to determine whether *C. jejuni* and *C. coli* were positive for each sample. Two ceca samples (2.1%) were *C. jejuni* positive and 96 ceca samples (100%) were *C. coli* positive. In the case of crops, *Campylobacter jejuni* and *coli* was detected in 11 (13.1%) and 61 (72.6%) samples, respectively. All ceca had *C. jejuni* or *C. coli* while 12 crops (14.3%) did not yield both *C. jejuni* and *C. coli* (Wesley et al., 2005).

Bily et al. (2010) evaluated *Campylobacter* spp. carriage in cecal contents of turkeys both quantitatively and qualitatively, during and following the slaughtering process. For quantitative analysis, samples were directly plated on Karmali plates. For qualitative analysis, samples were enriched in Preston broth and then enriched samples were streaked onto a Virion medium with agar and blood. Four turkey flocks were sampled in four different months (July, September, October, and November) and 30 ceca were collected at post-evisceration from each flock. Most of the ceca were positive for *Campylobacter* with all ceca collected in July, October, and November being *Campylobacter* positive (100%) and their contamination levels were 6.0, 7.2, and 6.0 log CFU/g, respectively. Ceca samples collected in September indicated the lowest *Campylobacter* prevalence and contamination level; the detection rate was 73.3% and average population was 2.1 log CFU/g (Bily et al., 2010).

Van Gerwe et al. (2010b) compared *Campylobacter* counts in ceca (*n* = 24) and crops (*n* = 23) of broilers of 31 days of age with a direct plating method using modified charcoal cefoperazone deoxycholate agar plates and detected an average 8.5 and 4.8 log CFU/g in ceca and crop, respectively (Van Gerwe et al., 2010b). When Yusrizal and Chen (2003) investigated the effects of inulin obtained from chicory roots on *Campylobacter* they enumerated *Campylobacter* in various organs including gizzard, small intestine, large intestine, cecal intestine and feces of the broiler chickens. *Campylobacter* populations in GIT from control chickens without inulin treatment revealed contamination levels in lower GIT that were higher than the upper GIT while *Campylobacter* populations in the large intestine (5.3 and 5.4 log CFU/g for male and female, respectively) and cecal intestine (5.3 and 4.4 log CFU/g for male and female, respectively) were relatively higher than the gizzard (2.5 and 2.5 log CFU/g) and small intestine (3.6 and 3.8 log CFU/g) (Yusrizal and Chen, 2003).

Most studies investigating *Campylobacter* prevalence in GIT have included ceca as the primary GIT organ of focus and the majority of these studies reported that poultry ceca had a greater percentage of *Campylobacter* (at least 73.3% positive) (Musgrove et al., 2001; Wesley et al., 2005; Bily et al., 2010) except for one study that resulted in only 3.8% *Campylobacter* positive birds (Byrd et al., 1998). The prevalence of *Campylobacter* was also generally high in poultry crops (Byrd et al., 1998; Musgrove et al., 2001). According to the study performed by Wesley et al. (2005), both ceca and crops of turkey were predominantly contaminated with *C. coli* rather than *C. jejuni* (Wesley et al., 2005). In terms of *Campylobacter* populations, their counts were generally higher in ceca than crop. In most studies more than 5 log CFU/g *Campylobacter* in ceca occurred and these population levels were generally greater when compared with populations enumerated from crops (Musgrove et al., 2001; Yusrizal and Chen, 2003; Bily et al., 2010; Van Gerwe et al., 2010b). These results suggest that the cecum appears to be a preferred habitat for *Campylobacter* but the proportion of *Campylobacter* versus total GIT microbial populations in each respective GIT compartment would need to be determined to confirm this. Consequently, even if the ceca is a primary target for the GIT, mitigation strategies directed toward other GIT compartments may be important and should be considered for mitigation as well, particularly if the lower GIT *Campylobacter* colonization is influenced by the appearance of *Campylobacter* in the upper GIT.

## Prebiotics – General Concepts

Prebiotics were initially defined in 1995 as ‘non-digestible food ingredients that have a beneficial effect on the host by selectively stimulating already existing bacterial species’s growth and/or activity in the colon, therefore attempt to improve host health’ (Gibson and Roberfroid, 1995). Prebiotics can reach the lower intestine and become accessible for GIT indigenous microbiota and subsequent stimulation of growth of specific bacterial groups considered “beneficial” leading to the production of short chain fatty acids and other fermentation products (Al-Sheraji et al., 2013). As defined by Roberfroid (2007) ideal prebiotics should (1) withstand exposure to gastric acid, hydrolysis by mammalian enzymes, and gastrointestinal absorption; (2) be fermented by intestinal microbiota; and (3) selectively stimulate the growth and/or activity of colon bacteria that are helpful to the host. Instead of only focusing on colonic bacteria, Gibson et al. (2004) generalized the definition of prebiotics which ‘are selectively fermented ingredient that represent particular changes in the gastrointestinal microflora’s composition and/or activity that confers benefits on host wellbeing and health.’

Research efforts to understand and interpret interactions between microbiota of the intestine and prebiotic substrates increased dramatically with the advent of community-wide sequencing, thus becoming essential to achieve consensus regarding the most appropriate definition of a prebiotics among the scientific community (Hutkins et al., 2016). The definition of prebiotics has been expanded to include non-carbohydrate substances and to deliver health benefits to host body sites other than the GIT (Gibson et al., 2017). With increased knowledge of interactions between the GIT microbiota and prebiotics, the classification of prebiotics has shifted to include a wide range of non-digestible oligosaccharides with varying carbon chain length and not being digestible by the host (Ricke, 2018). This reframing of prebiotic sources in turn influences approaches for identifying prebiotic candidates for use in poultry, their respective benefits on poultry health and the potential for limiting *Campylobacter* colonization in the avian GIT.

## Prebiotics to Improve GIT Health of Poultry

Various prebiotics have been applied to poultry feed with mannan-oligosaccharides, inulin and its hydrolysate (fructooligosaccharides), and xylooligosaccharides being some of the more common prebiotic sources that have been examined for inclusion in poultry diets. Most of the research focus has been on the impact these various prebiotics have on reducing pathogen colonization in poultry. In some cases effort has been made to identify whether certain groups of GIT bacteria considered beneficial to the bird are increased by the presence of prebiotics in the diet. The impact on beneficial GIT bacteria along with the production of specific fermentation products has been suggested as having potential positive impacts on the bird host.

Mannan-oligosaccharides, derived from the yeast cell wall, are comprised of mannose, glucan, proteins, and phosphate radicals that are linked by β-1,4 glycosidic bonds (Klis et al., 2002). The fact that poultry do not have enzymes for metabolizing mannan-oligosaccharides ensures that they reach the GIT without any host derived enzymatic digestion (Pourabedin and Zhao, 2015). Using mannan-oligosaccharides helps to reduce pathogenic bacteria by interference with attachment via the type-1 fimbriae found on many Gram-negative bacteria (e.g., *Escherichia coli* and *Salmonella*) (Ferket, 2004). Those particular bacteria and mannose-based oligosaccharides can bind together through the lectin, therefore mannan-oligosaccharides can reduce attachment of the corresponding pathogen to the gut (Oyofo et al., 1989; Spring et al., 2000; Ferket, 2004). Supplementation of mannan-oligosaccharides in feed diets modulated cecal microbial content of broilers by markedly reducing the number of *Clostridium perfringens*, which is of great significance as a poultry pathogen (Jamroz et al., 2004; Yang et al., 2008), and selecting for the growth of beneficial bacteria for host health such as *Bifidobacteria* spp. and *Lactobacillus* spp. which are generally viewed as GIT bacteria capable of eliciting positive impacts on the host (Baurhoo et al., 2007).

Inulin is a fructan connected by β (2-1) glycosidic bond extracted from chicory (*Cichorium intybus*) (Phelps, 1965; Niness, 1999). Supplementation of inulin in poultry feed is believed to modulate intestinal microbiota resulting in the proliferation of beneficial bacteria including *Lactobacillus* and *Bifidobacteria* while simultaneously inhibiting pathogenic bacteria such as *Escherichia coli, C. perfringens*, or *Staphylococcus aureus* (Nabizadeh, 2012; Lopes et al., 2013; Buclaw, 2016). Microbiota populations in the cecal contents of chickens were altered by addition of inulin in feed, with increasing numbers of *Bifidobacteria* and decreasing the populations of *E. coli* (Nabizadeh, 2012). Some bifidobacterial species can suppress Gram-positive and Gram-negative pathogens including *Salmonella, Campylobacter*, and *E. coli* (Gibson and Wang, 1994; Samanta et al., 2012). The activities of enzymes derived from gut microbiota of rats and humans were affected by the pH level (as the pH level increased, Beta – glucosidase activity was diminished) (Mallett et al., 1989) and the pH level of the cecal contents notably decreased when 1% inulin was added to feed for broiler chickens (Nabizadeh, 2012).

Arabinoxylans are present in cereal fibers and mainly consist of two pentose sugars- arabinose and xylose (James et al., 2003). Hydrolytic degradation of the heteropolymer arabinoxylans generates arabinose-substituted xylooligosaccharides and non-substituted xylooligosaccharides (Broekaert et al., 2011). Xylooligosaccharides are xylopyranoise units which are linked by β-1,4 linkages (Carvalho et al., 2013). Broilers possess only limited enzymatic capabilities to hydrolyze the linkage between xylose thus xylooligosaccharides can arrive relatively intact to the cecum and the lower intestinal tract (Pourabedin and Zhao, 2015). Supplementation of feed with xylooligosaccharides (2 g xylooligosaccharides/kg diet) increased the proportion of *Lactobacillus* in the cecum (Pourabedin et al., 2015). In the ceca of broilers supplemented by xylooligosaccharides in feeds, significantly higher abundance of *Lactobacillus crispatus* and *Anaerostipes butyraticus* was observed with xylooligosaccharides supplementation and higher gene copies of butyryl-CoA:acetate-CoA transferase, which is an enzyme in a butyrate production pathway in the gut, were also detected (Duncan et al., 2004; De Maesschalck et al., 2015). Butyrate is considered beneficial to gastrointestinal function (De Maesschalck et al., 2015) and it can also improve growth performance of animals and modulate the microbiota composition and metabolic activity in the intestine (Guilloteau et al., 2010; Canani et al., 2011).

## Prebiotic Supplementation to Limit *Campylobacter* in Conventionally Raised Poultry

While prebiotics appear to be generally effective to limit establishment of foodborne pathogens in the GIT and improve overall GIT health, less is known about their impact directly on *Campylobacter* levels in the avian GIT. Several studies have examined the influence of prebiotics as feed additives on *Campylobacter* populations in GIT of poultry (Fernandez et al., 2000; Yusrizal and Chen, 2003; Baurhoo et al., 2009; Arsi et al., 2015; Guyard-Nicodeme et al., 2015; Rezaei et al., 2015; Park et al., 2017a). The majority of the *in vivo* studies reported reductions on *Campylobacter* counts or relative abundance in cecal contents and other intestinal sections of the chicken GIT (Fernandez et al., 2000; Yusrizal and Chen, 2003; Baurhoo et al., 2009; Arsi et al., 2015; Guyard-Nicodeme et al., 2015) while a few studies did not observe significant changes in *Campylobacter* populations and relative abundance when prebiotics were supplemented in an attempt to limit *Campylobacter* compared to control birds not fed prebiotics (Rezaei et al., 2015; Park et al., 2017a). Table 2 lists some of the studies examining prebiotics as feed additives on *Campylobacter* counts in poultry GIT *in vivo*.

**Table 2:** Impact of prebiotics as feed additive on *Campylobacter* counts in poultry gastrointestinal tract.

Prebiotic treatment	Tested GIT	*Campylobacter* test method	Changes in *Campylobacter* counts/relative abundance	Reference
Feed with 0.1% xylanase (Avizyme-1300)	Cecal, sall intestine, and large intestine	Direct plating	*Campylobacter* counts were reduced by xylanase supplemented diet in cecal, small intestine, and large intestine	Fernandez et al., 2000
Feed with 1.0% inulin (Raftifeed^®^IPF) Feed with 1.0% oligofructose (Raftifeed^®^OPS)	Fecal, gizzard, small intestine, large intestine, and cecal intestine	Direct plating	*Campylobacter* counts were reduced by inulin or oligofructose supplemented diet in the large and cecal intestine No significant changes in *Campylobacter* counts in fecal microflora, gizzard contents, small intestine compared to control	Yusrizal and Chen, 2003
Feed with 0.2% mannan-oligosaccharides Feed with 0.5% mannan-oligosaccharides	Cecal intestine	Direct plating	*Campylobacter* counts were reduced by 0.2% mannan-oligosaccharide supplemented diet in cecal intestine No significant reductions in 0.5% mannan-oligosaccharide	Baurhoo et al. (2009)
Feed with 0.2% Biolex^®^MB40 Feed with 0.2% Leiber^®^ExCel	Cecal intestine	Direct plating	*Campylobacter* counts were reduced by both prebiotics supplemented diet in cecal intestine	Park et al., 2014
Probiotics (isolate 1, 2, or 3) + prebiotics (0.125, 0.25, or 0.5% fructooligosaccharide or 0.04, 0.08, or 0.16% manna-oligosaccharides)	Cecal intestine	Direct plating	*Campylobacter* counts were not reduced by fructooligosaccharide or manna-oligosaccharide only *Campylobacter* counts were reduced by combination of probiotics and prebiotics in cecal intestine	Arsi et al., 2015
Feed with 0.125% prebiotic-like product (Original XPC^TM^)	Cecal intestine	Direct plating	*Campylobacter* counts were reduced by prebiotic-like product supplemented diet in cecal intestine	Guyard-Nicodeme et al., 2015
Feed supplemented with 0.5 and 1% oligosaccharides extract from palm kernel expeller (OligoPKE)	Cecal intestine	Quantitative real time PCR	No significant changes in *Campylobacter* counts in cecal contents by oligosaccharides compared to control	Rezaei et al., 2015
Feed with 0.2% β-glucan and mannan-oligosaccharides (Biolex^®^MB40)	Cecal intestine	Next generation sequencing with Illumina MiSeq	No significant changes in the relative abundance of *Campylobacter* compared in cecal contents by β-glucan and mannan-oligosaccharides compared to control Significant lower *Campylobacter* abundance in prebiotic treated group compared to antibiotic treated group	Park et al., 2017a
Feed with 0.1% plum fibers Feed with 0.1% fructooligosaccharides Feed with 0.2% galactooligosaccharides	Cecal intestine	Direct plating	No significant changes in *Campylobacter* counts in cecal contents by prebiotics compared to control	Park et al., 2017b


Adding 1.0% inulin (Raftifeed^®^IPF) which was obtained from chicory roots extraction with hot water resulted in significant reduction of *Campylobacter* counts in large intestine contents from 42 day old female broilers; 3.8 log CFU/g of *Campylobacter* by inulin supplement versus 5.4 log CFU/g in control birds (Yusrizal and Chen, 2003). Although there were no significant differences, *Campylobacter* populations in the large intestine contents from male broilers fed inulin supplements were lower than control birds (4.4 log CFU/g versus 5.3 log CFU/g) (Yusrizal and Chen, 2003). Birds given feed containing 1.0% oligofructose (Raftifeed^®^OPS) (a partial enzymatic hydrolysate of chicory inulin) exhibited significant reductions in *Campylobacter* counts in the large intestinal contents of 42-day old female and male broilers; 3.69 CFU/g (oligofructose) versus 5.3 log CFU/g (control) in male broilers and 4.1 log CFU/g (oligofructose) versus 5.4 log CFU/g (control) in female broilers (Yusrizal and Chen, 2003). Oligofructose also decreased *Campylobacter* counts in the cecal contents of 42-day old male broilers (3.3 log CFU/g vs. 5.3 log CFU/g). However, there were no significant changes in *Campylobacter* populations in the fecal, gizzard, and small intestine contents from birds fed both inulin and oligofructose (Yusrizal and Chen, 2003).

Baurhoo et al. (2009) investigated the effects of diets supplemented with mannan-oligosaccharide or antibiotics on *Campylobacter* colonization of broiler chicken ceca. They tested an antibiotic free diet (control), a diet with commonly used antibiotics (virginiamycin and bacitracin), and a diet containing mannan-oligosaccharide administered at different concentrations (0.2 and 0.5%). They enumerated *Campylobacter* populations in cecal contents from birds at 14, 24, and 34 days of age using *Campylobacter* agar base with lysed horse blood, Preston *Campylobacter* selective supplement, and *Campylobacter* growth supplement (Baurhoo et al., 2009). The addition of mannan-oligosaccharide to the feed resulted in a significant decrease of *Campylobacter* counts in birds fed 0.2% supplemented feed while there were no significant changes at day 34 in birds fed 0.5% supplemented feed when compared to control birds (Baurhoo et al., 2009). This lack of a dosage response is consistent with the conclusion of the authors that there were generally no additional GIT health benefits when mannan-oligosaccharide levels were increased from 0.2 to 0.5%. It is not clear mechanistically why additional mannan-oligosaccharide would not further decrease *Campylobacter* levels. However, increased *Bifidobacteria* were only detected in the ceca of 0.5% supplemented birds versus 0.2% fed birds at day 24 and not day 34, while cecal lactobacilli were not statistically different between 0.2 and 0.5% supplemented birds on either day 24 or 34. The authors noted that mannan-oligosaccharides are believed to competitively inhibit GIT colonization of pathogens such as *Campylobacter* by binding to their mannose specific type 1 fimbriae but it should be noted that they did not see a statistical difference in litter concentrations of *Campylobacter* for any of the days sampled (days 14, 24, and 34) for either 0.2 or 0.5% mannan-oligosaccharides. Further studies involving more intermediate increments of mannan-oligosaccharides will be needed to delineate whether dosage responses in birds are possible with this prebiotic.

There have also been studies where no impact on *Campylobacter* occurred in chicken cecal contents when prebiotics or prebiotic-like compounds were included (Rezaei et al., 2015; Park et al., 2017a,b). Rezaei et al. (2015) tested three dietary treatments including basal diet as control and basal diet supplemented with 0.5 and 1% oligosaccharides (extract from palm kernel expeller, OligoPKE) and enumerated *Campylobacter* counts in cecal contents at day 21 and 35 broiler chickens with quantitative real time PCR; resulting in no significant changes in *Campylobacter* enumerated populations following prebiotic addition.

Park et al. (2017a) applied next generation sequencing technology based on an Illumina MiSeq platform to investigate microbiological compositions in the cecal contents of 1, 2, 4, and 6 weeks old chickens fed either a basal diet, a diet with antibiotic (consisting bacitracin methylene disalicylate and bacitracin, BMD50), or a diet with a yeast-based prebiotic (consisting 1,3-1,6-β-D-glucan and mannan-oligosaccharides derived from the cell walls of *Saccharomyces cerevisiae*, Biolex^®^MB40; Leiber GmbH, Hafenstrase, Germany). When compared to the control group fed the basal diet, the treatment group fed with prebiotic exhibited similar *Campylobacter* levels. However, a significantly lower *Campylobacter* abundance was observed compared to treatment group fed with antibiotics at 4 weeks of age. *Lactobacillus* abundance was significantly lower in the antibiotic treated group compared to the control and prebiotic fed groups. Successfully predicting the potential for success or failure of a particular prebiotic to mitigate foodborne pathogens such as *Campylobacter* will require further in-depth delineation of the mechanisms associated with prebiotics and the GIT microbiota. This will no doubt require not only next generation sequencing to identify specific taxa but the metabolic activities of the resident GIT microbial populations in the presence of particular prebiotics and how changes may impact *Campylobacter* either directly or indirectly.

## Prebiotic Supplementation to Limit *Campylobacter* in Non-Conventionally Raised Poultry

Most of the research on prebiotics and poultry have been conducted with birds raised under conventional commercial housing conditions or at least experimental environments that attempted to simulate these types of conditions (Park et al., 2013; Ricke, 2015). Less work has been done with poultry raised under free range or pasture flock settings. However, the choices in feed additives are considered more restrictive for birds raised under these antibiotic – free conditions even though there is probably more of a need for feed additives to reduce exposure of these birds to a wide range of pathogens (Park et al., 2013). As this industry expands there is a clear need for development of feed additives that are not only acceptable but amendable to routine supplementation. Prebiotics, depending on their source and how they are generated, could certainly be a possibility.

Park et al. (2014) tested the effects of two commercial prebiotics, Biolex^®^MB40 and Lieber^®^ExCel derived from brewer’s yeast cell walls, when added to feed provided to pasture raised naked neck broilers. *Campylobacter* counts recovered from the cecal contents were assessed using Campy-Cefex plates. The populations of *Campylobacter* in the control group were 6.49 log CFU/100 mg and both treatment groups exhibited significantly lower counts (6.07 log CFU/100 mg for both groups) than control birds (Park et al., 2014). While statistically significant it remains unclear whether these biologically slight differences in cecal *Campylobacter* would impact *Campylobacter* levels of the processed birds or be relevant for incidence of human campylobacterosis. Given the relationship between the GIT microbiota and *Campylobacter*, denatured gradient gel electrophoresis (DGGE) was also employed in this study to detect changes in the cecal microbiota in birds fed the different treatments. Banding patterns of DGGE recovered from the birds were generally similar among all treatments but certain specific bands were specifically identified with a particular group and banding intensities also differed in groups shared among the treatment groups. Isolation and sequencing of specific bands of interest using an ABI 3100 capillary analyzing system (Applied Biosystems) revealed *Bacteroides salanitronis* occurring in all groups and *Barnesiella viscericola* and *Firmicutes* identified from bands associated with both treatments. Interestingly based on band sequencing three *Campylobacter* species were identified, namely, *C. jejuni, C. coli*, and *C. lari*.

Clearly, as Park et al. (2014) noted, DGGE-based methods suffer from several limitations that handicap comprehensive interpretation. More in-depth assessment of the pasture flock cecal microbiome response has since become possible with the introduction of next generation sequencing of 16S rRNA genes. Park et al. (2017b) evaluated the effects of prebiotics when they were supplemented to feed such as 0.1% plum fibers, 0.1% fructooligosaccharides, and 0.2% galactooligosaccharides to broiler chickens. *Campylobacter* levels in cecal contents were enumerated with a plating method using Campy-Line agar and the cecal microbiome was also evaluated with next generation sequencing using an Illumina HiSeq platform. With the direct plating method, there were no significance differences among all groups (control and three treatment groups) (Park et al., 2017b). Using synthetic learning in microbial ecology (SLiME) analysis, they found that feed supplements modulated the diversity of microbiota and operational taxonomic units identified as belonging to the genus *Alistipes* and *Lactobacillus intestinalis* were indicated as a potential predictive feature for *Campylobacter* populations (>10% and >25% increase mean squared error, respectively) suggesting a potential metabolic interaction (Park et al., 2017b). However, as Park et al. (2017b) point out, age of the bird was also highly related to the pattern of appearance for both *Campylobacter* and these two non-*Campylobacter* bacteria and consequently some of this apparent relatedness could be coincidental. Some of this may become more clear as the resolution of bioinformatic tools continues to improve to the point that specific factors can be delineated as to their respective contributions.

## Prebiotics Combined With Other Feed Additives

While there has been extensive research focused on prebiotics administered as stand alone feed amendments, efforts have been undertaken to evaluate them in the presence of other GIT modulators. An obvious combination would be to combine them with specific probiotic cultures of bacteria that could use the corresponding prebiotic as a substrate. The resulting synbiotics are the combination of prebiotics and probiotics that in theory would select and help to maintain probiotic sustainability in the GIT (Collins and Gibson, 1999). At the beginning of the 20th century Elie Metchnikoff, commonly considered a pioneer of modern probiotics, initially observed enhancement of health and longevity in humans upon the regular consumption of lactic acid bacteria and consequently hypothesized that lactic acid bacteria could replace or diminish the number of putrefactive bacteria in the gut (Anukam and Reid, 2007). The concept of probiotics was introduced in 1965 as ‘substances secreted by single microorganism which stimulates others growth’ (Lilly and Stillwell, 1965). The term ‘probiotics’ gained general acceptance and its definition was further refined; Fuller (1989) defined probiotics as follows: ‘a live microbioal feed supplement beneficially affects the host by improving its intestinal microbiota’ (Fuller, 1989). *Lactobacilli* spp., *Bifidobacteria* spp., and Gram-positive cocci including *Enterococcus faecium, Streptococci diaacetylactis* are common examples of probiotics (Collins and Gibson, 1999). Ideal probiotics should (1) not have toxic substances or be pathogens; (2) resist gastric acid and bile from the liver thus survive in the intestinal tract; (3) attach themselves to epithelial tissue; (4) produce inhibitory compounds against pathogens; (5) stimulate the immune system; and (6) alter microbial activities (Fuller, 1989; Gibson and Fuller, 2000; Rolfe, 2000; Simmering and Blaut, 2001; Patterson and Burkholder, 2003). However, the effect of using probiotics could be transient because of the difficulty to achieve continuous adherence and/or retention in the GIT (Goldin and Gorbach, 1984; Jonsson, 1986). Given the considerable complexity and large numbers of indigenous bacteria already predominating the GIT prior to application of probiotics, it is not surprising that continuous feeding may be necessary to prolong their efficacy (Fuller, 1989). Likewise, the extensive shifting in the composition and subsequent diversity of the cecal microbiota as birds age could play a key role in the respective efficacy of different prebiotics and pathogen colonization (Park et al., 2017c; Ricke, 2018).

The impact of the combination of probiotics and prebiotics (synbiotics) on *Campylobacter* counts was investigated by Arsi et al. (2015). Feed additives included strains isolated from healthy chickens (isolate 1: *Bacillus* spp., isolate 2: *Lactobacillus salivarius* subsp. *salivarius*, and isolate 3: *Lactobacillus salivarius* subsp. *salicinius*) and/or a prebiotic with different concentrations (0.125, 0.25, or 0.5% fructooligosaccharide or 0.04, 0.08, or 0.16% mannan-oligosaccharide) (Arsi et al., 2015). *Campylobacter* populations were enumerated with direct plating on Campy-Line agar. Individual treatments of either prebiotic or probiotic did not result in detectable reductions of *Campylobacter* population in cecal contents. However, their combination resulted in a considerable level of reduction; isolate 3 + 0.04% mannan-oligosaccharide in feed exhibited a 3.3 log reduction compared to control (Arsi et al., 2015). The authors presumed that this may be attributed to the promotion of the growth of probiotics by mannan-oligosaccharide thus resulting in the concomitant reduction of *Campylobacter* in the cecal contents.

Non-probiotic feed additives have also been combined with prebiotics in poultry studies. For example, Guyard-Nicodeme et al. (2015) evaluated efficacy of various commercially available feed additives containing organic acid, short chain fatty acids, monoglycerides, plant extracts, prebiotic-like compounds, and probiotics (Lacto-butyrin, Biotronic^®^Top3 Campylostat, Admix^®^Precision, Excential Butycoat, Power Protexion^®^, Excential Alliin Plus, Anta^®^Phyt, Calsporin^®^, Ecobiol^®^, PoultryStar^®^, and Original XPC^TM^). Chicks were fed either a control diet or diets supplemented with feed additives (total of 13 groups) and *Campylobacter* populations were enumerated in ceca of broilers at 14, 35, and 42 days of age by direct plating on modified Charcoal cefoperazone deoxycholate agar (Guyard-Nicodeme et al., 2015). Prebiotic-like compounds (Original XPC^TM^) consisting of post-fermentation growth medium residues, residual yeast cells, and yeast cell wall fragments (mannan-oligosaccharides and β-glucans) yielded no significant reductions of *Campylobacter* at 14 and 35 days of age compared to control while a 3.2 log reduction were obtained in birds at 42 days of age. This level of reduction was greatest among the 12 tested feed additive with 2.1 and 1.7 log reductions being observed in the presence of the Admix^®^Precision and Calsporin^®^treatments, respectively, while the other 9 feed additives exhibited negligible bactericidal effects in birds at 42 days of age (Guyard-Nicodeme et al., 2015). Before comprehensive overall conclusions can be drawn from such results further studies will need to be conducted to more quantitatively separate individual feed amendment treatments impacts on bird GIT health, performance and pathogen levels and link these to mechanism(s) associated with specific additives. This will require a number of independent trials to be conducted over a wide range of management practices along with in-depth profiling of GIT responses using molecular methods to characterize the GIT microbial populations and detailed GIT lumen metabolite and host GIT tissue responses.

Given that some prebiotic sources are not only indigestible but can occur as complex polymers, combining them with active feed grade enzymes has merit for increasing efficacy (Ricke, 2018). Consequently, combining enzymes with prebiotics has been suggested as a means for enhancing the endogenous enzyme production in birds, aid digestion of fiber components, render nutrients for easier digestion, reduce the effects of antinutritional factors, and raise efficacy in feed formulation (Ferket, 1993). Limited studies have been conducted to examine the impact of such combinations and *Campylobacter* occurrence. Fernandez et al. (2000) assessed the *C. jejuni* population levels in the small intestine, cecum, and large intestines of broiler chickens fed a wheat diet supplemented with a xylanase (control: wheat and maize-based feeds). They used a marker strain of *C. jejuni* with resistance to nalidixic acid and *Campylobacter* populations in the intestine were determined using a direct plating method on *Campylobacter* blood-free selective agar with ceforperazone selective supplement and nalidixic acid. Minimal differences in *C. jejuni* were obtained in the small and large intestines of chicken fed the 0.1% xylanase-supplemented diet (1.4 and 2.3 log CFU, respectively) compared to controls (wheat-based: 1.7 and 2.4 log CFU; maize-based: 1.5 and 2.4 log CFU, respectively). However, there were statistically less *C. jejuni* in the ceca of birds fed the wheat-xylanase combination (2.3 log CFU, respectively) compared to birds fed wheat and maize-based diets (wheat-based: 2.8, log CFU; maize-based: 2.6 log CFU, respectively) without xylanase which corresponded to a significant decrease in jejunum viscosity in the enzyme supplemented birds (Fernandez et al., 2000). How, biological relevant these relatively small reductions would be to achieve consistent overall *Campylobacter* reduction in commercial operations remains to be determined.

## Future Directions for Prebiotic Application to *Campylobacter* Control in Chickens

While it appears that the majority of the poultry studies conducted thus far on *Campylobacter* colonization and response to prebiotics indicate some level of reduction, the results remain variable. Some of this may be related to differences in methodologies used to detect and quantitate *Campylobacter* recovered from the poultry GIT. Typically, *Campylobacter* counts are enumerated by direct plating on selective medium from collected intestinal contents and in some cases culture independent methods such as quantitative PCR and next generation sequencing technology are also employed (Table 2). However, culture-based methods may not always accurately reflect actual *Campylobacter* populations. For example, Kim et al. (2017) sequenced pooled colonies recovered from Campy-Cefex selective media which had been the selective media recommended by USDA at the time for *Campylobacter* isolation from chicken carcass rinsates using an Illumina MiSeq flatform. Based on the 16S rDNA microbiome sequencing the *Campylobacter* selective media apparently supported growth of a mixed background bacterial population, some of which were identified as *Campylobacter* but others were also be recovered such as *Clostridiaceae, Paenibacillus, Lactobacillus, Bacillaceae, Acinetobacter, Enterobacteriaceae, Bacillus, Planococcaceae, Clostridium, Enterococcus*, and *Sporanaerobacter* (Kim et al., 2017). Intuitively it would be anticipated that choice of selective media could be influenced and potentially biased for recovering *Campylobacter* from the highly diverse poultry GIT. The growth of these non-*Campylobacter* background bacteria could cause lower accuracy of the media for enumerating *Campylobacter* and in turn, assessing the impact of feed additives such as prebiotics. Therefore, other culture independent methods such as qPCR could be an effective alternative method to culture based methods with plating on selective media to overcome some of the issues associated with culture-based methods.

Moreover, technical progress in the advent of high-throughput sequencing technology and the corresponding bioinformatic tools will lead to obtaining comprehensive information of the microecology of a variety of samples including the avian GIT (Land et al., 2015; De Filippis et al., 2018). Since prebiotics are well known to have an impact on the gut microbiota and pathogenic bacteria leading to detectable shifts in gut microbial populations, a series of research approaches involving high-throughput sequencing technologies and bioinformatics to evaluate changes on microbiological compositions in the GIT by prebiotics could be invaluable for achieving a better understanding of the mechanism(s) associated with prebiotics on limiting pathogens including *Campylobacter* (Cummings and Macfarlane, 2002; Edens, 2003; Yang et al., 2009; Hajati and Rezaei, 2010).

## Conclusion

In the present review, *Campylobacter* in the GIT was briefly discussed, followed by prebiotics and their general effects on the intestine and finally recent research studies aimed at providing empirical data on prebiotic efficacy to reduce *Campylobacter* counts/abundance in poultry intestine. Based on the limited research discussed here, addition of prebiotics to the poultry feed generally appeared to result in detectable decreases in *Campylobacter* populations in the different regions of poultry GIT; indicating that the potential ability of prebiotics as feed supplements to limit *Campylobacter* in the poultry GIT does exist. Whether these colonization reductions in the chicken GIT specifically associated with feeding prebiotics are sufficient to result in reduction of human campylobacterosis cases remains to be determined. Only limited research data is available on *Campylobacter* reduction in poultry by prebiotics in large scale commercial trials currently. In conjunction with devising more focused empirical research strategies on the efficacy of prebiotics, examination under typical poultry husbandry conditions is also needed to develop practical recommendations for their use as feed supplements. Along these lines, other important factors such as bird performance and organoleptic meat quality should also be considered along with *Campylobacter* reduction.

Mitigating *Campylobacter* in poultry will continue to be an important focus for the poultry industry. Opportunities exist for accomplishing some control at the pre-harvest poultry production level. However, this clearly requires gaining a better understanding of the interaction between the poultry GIT microbial community and the resident *Campylobacter* species that co-exist. Prebiotics could be a promising strategy to control *Campylobacter* in poultry but definitive recommendations will require in-depth characterization of the poultry GIT microecology and the role the host plays in influencing that ecology. This will require more of an “omics” approach that encompasses not only microbiota sequencing but metabololic profiling as well host GIT tissue metabolic enzyme and immune responses. In addition, understanding *Campylobacter* behavior in the avian GIT tract at the molecular level may be critical as well given its close association with indigenous GIT microbiota. Combining the information generated from the various approaches should help to optimize prebiotic choice and application for practical mitigation of *Campylobacter* in the avian GIT.

## Author Contributions

All authors listed have made a substantial, direct and intellectual contribution to the work, and approved it for publication.

## Conflict of Interest Statement

HP is employed by the company Diamond V. The remaining authors declare that the research was conducted in the absence of any commercial or financial relationships that could be construed as a potential conflict of interest.
